# Supplementary material for: Comparison of alignment and spondylolysis fracture angle in bilateral and unilateral spondylolysis
Source: PLoS One. 2022 Oct 18;17(10):e0276337. doi: 10.1371/journal.pone.0276337 (PMC9578603; doi:10.1371/journal.pone.0276337)
Supplement: Attachment — Submitted filename: Response_to_Reviewers.docx [file pone.0276337.s003.docx]

September 23, 2022

Editorial Board

*PLOS ONE*

Manuscript Number: PONE-D-22-11206

“Comparison of alignment and spondylolysis fracture angle in bilateral and unilateral spondylolysis” by Kanta Matsuzawa

Dear Editor:

Thank you for your letter regarding our submission. We are grateful for the detailed feedback provided by the reviewers, which we feel has helped us significantly improve the paper. Attached are our point-by-point responses to the reviewers’ comments and our revised manuscript, which we hope will now meet your approval. We believe that our revisions have addressed the issues raised by the reviewers and trust that the manuscript is now suitable for publication in *PLOS ONE.* Thank you again for your thoughtful comments, and we look forward to hearing from you soon.

Sincerely,

Kanta Matsuzawa

**Journal Requirements:**

**Please review your reference list to ensure that it is complete and correct. If you have cited papers that have been retracted, please include the rationale for doing so in the manuscript text, or remove these references and replace them with relevant current references. Any changes to the reference list should be mentioned in the rebuttal letter that accompanies your revised manuscript. If you need to cite a retracted article, indicate the article’s retracted status in the References list and also include a citation and full reference for the retraction notice.**

→We have not changed the references. We modified only the format of references.

1.	Sakai T, Sairyo K, Suzue N, Kosaka H, Yasui N. Incidence and etiology of lumbar spondylolysis: review of the literature. Journal of Orthopaedic Science. 2010;15:281-288. doi: 10.1007/s00776-010-1454-4, PubMed PMID: 20559793.

2.	Randall RM, Silverstein M, Goodwin R. Review of pediatric spondylolysis and spondylolisthesis. Sports Medicine and Arthroscopy Review. 2016;24:184-187. doi: 10.1097/JSA.0000000000000127, PubMed PMID: 27811518.

3.	Grazina R, Andrade R, Santos FL, Marinhas J, Pereira R, Bastos R, et al. Return to play after conservative and surgical treatment in athletes with spondylolysis: A systematic review. Physical Therapy in Sport : official journal of the Association of Chartered Physiotherapists in Sports Medicine. 2019;37:34-43. doi: 10.1016/j.ptsp.2019.02.005, PubMed PMID: 30826586.

4.	Cooper G. Non-operative treatment of the lumbar spine. 2015.

5.	Sairyo K, Katoh S, Sasa T, Yasui N, Goel VK, Vadapalli S, et al. Athletes with unilateral spondylolysis are at risk of stress fracture at the contralateral pedicle and pars interarticularis: a clinical and biomechanical study. The American Journal of Sports Medicine. 2005;33(4):583-590. doi: 10.1177/0363546504269035, PubMed PMID: 15722292.

6.	El Rassi G, Takemitsu M, Glutting J, Shah SA. Effect of sports modification on clinical outcome in children and adolescent athletes with symptomatic lumbar spondylolysis. American Journal of Physical Medicine and Rehabilitation. 2013;92:1070-1074. doi: 10.1097/PHM.0b013e318296da7e, PubMed PMID: 24141103.

7.	Roussouly P, Gollogly S, Berthonnaud E, Labelle H, Weidenbaum M. Sagittal alignment of the spine and pelvis in the presence of L5-s1 isthmic lysis and low-grade spondylolisthesis. Spine (Phila Pa 1976). 2006;31(21):2484-2490. doi: 10.1097/01.brs.0000239155.37261.69, PubMed PMID: 17023859.

8.	Sugawara K, Iesato N, Katayose M. Segmental lordosis of the spondylolytic vertebrae in adolescent lumbar spondylolysis: differences between bilateral L5 and L4 spondylolysis. Asian Spine Journal. 2018;12:1037-1042. doi: 10.31616/asj.2018.12.6.1037, PubMed PMID: 30322253; PubMed Central PMCID: PMC6284115.

9.	Marty C, Boisaubert B, Descamps H, Montigny JP, Hecquet J, Legaye J, et al. The sagittal anatomy of the sacrum among young adults, infants, and spondylolisthesis patients. European Spine Journal. 2002;11(2):119-125. doi: 10.1007/s00586-001-0349-7, PubMed PMID: 11956917; PubMed Central PMCID: PMC3610511.

10.	Labelle H, Mac-Thiong JM, Roussouly P. Spino-pelvic sagittal balance of spondylolisthesis: a review and classification. European Spine Journal. 2011;20 Suppl 5:641-646. doi: 10.1007/s00586-011-1932-1,PubMed PMID: 21809015; PubMed Central PMCID: PMC3175928.

11.	Sairyo K. Spondylolysis fracture angle in children and adolescents on CT indicates the facture producing force vector : A biomechanical rationale. The Internet Journal of Spine Surgery. 2005;1:2.

12.	Nagamoto H, Abe M, Konashi Y, Kimura R, Takahashi M, Oizumi A. Rotation-related sports players demonstrate rotation-type lumbar spondylolysis fracture angle and decreased hip internal rotation range of motion. Journal of Orthopaedics. 2021;28:101-106. doi: 10.1016/j.jor.2021.11.010, PubMed PMID: 34898928; PubMed Central PMCID: PMC8640617.

13.	Matsumoto ME, Czerniecki JM, Shakir A, Suri P, Orendurff M, Morgenroth DC. The relationship between lumbar lordosis angle and low back pain in individuals with transfemoral amputation. Prosthetics and Orthotics International. 2019;43:227-232. doi: 10.1177/0309364618792746, PubMed PMID: 30122108.

14.	Terai T, Sairyo K, Goel VK, Ebraheim N, Biyani A, Faizan A, et al. Spondylolysis originates in the ventral aspect of the pars interarticularis: a clinical and biomechanical study. The Journal of Bone and Joint Surgery. 2010;92(8):1123-1127. doi: 10.1302/0301-620X.92B8.22883, PubMed PMID: 20675758.

**Reviewer #1:**

**1　Line 147: LLA was significantly larger. Line 149: Increase in LLA. Have already mentioned the above issue in the initial review. Besides the above mentioning, the authors have answered and revised thoroughly and worth publication in the journal.**

→Thank you for the suggestion. We have revised as suggested (Lines 147- ).

We measured LLA and SSA as alignment; LLA was significantly larger in all the spondylolysis patients than in the control group, and the LLA was significantly larger in bilateral than unilateral spondylolysis. Increase in LLA may lead to an increase in compressive and shearing forces on the pars interarticularis with lumbar extension [8].
